# Prolonged Exercise in Type 1 Diabetes: Performance of a Customizable Algorithm to Estimate the Carbohydrate Supplements to Minimize Glycemic Imbalances

**DOI:** 10.1371/journal.pone.0125220

**Published:** 2015-04-28

**Authors:** Maria Pia Francescato, Giuliana Stel, Elisabetta Stenner, Mario Geat

**Affiliations:** 1 Department of Medical and Biological Sciences, University of Udine, Udine, Italy; 2 Department of Laboratory Medicine, Ospedali Riuniti di Trieste, Trieste, Italy; Sapienza University of Rome, ITALY

## Abstract

Physical activity in patients with type 1 diabetes (T1DM) is hindered because of the high risk of glycemic imbalances. A recently proposed algorithm (named *Ecres*) estimates well enough the supplemental carbohydrates for exercises lasting one hour, but its performance for prolonged exercise requires validation. Nine T1DM patients (5M/4F; 35–65 years; HbA1c 54±13 mmol·mol^-1^) performed, under free-life conditions, a 3-h walk at 30% heart rate reserve while insulin concentrations, whole-body carbohydrate oxidation rates (determined by indirect calorimetry) and supplemental carbohydrates (93% sucrose), together with glycemia, were measured every 30 min. Data were subsequently compared with the corresponding values estimated by the algorithm. No significant difference was found between the estimated insulin concentrations and the laboratory-measured values (p = NS). Carbohydrates oxidation rate decreased significantly with time (from 0.84±0.31 to 0.53±0.24 g·min^-1^, respectively; p<0.001), being estimated well enough by the algorithm (p = NS). Estimated carbohydrates requirements were practically equal to the corresponding measured values (p = NS), the difference between the two quantities amounting to –1.0±6.1 g, independent of the elapsed exercise time (time effect, p = NS). Results confirm that *Ecres* provides a satisfactory estimate of the carbohydrates required to avoid glycemic imbalances during moderate intensity aerobic physical activity, opening the prospect of an intriguing method that could liberate patients from the fear of exercise-induced hypoglycemia.

## Introduction

Regular moderate-intensity physical activity is recommended in patients with type 1 diabetes (T1DM) [1–3] because it can reduce the risk of cardiovascular disease [4, 5] and it improves insulin sensitivity [6], although an independent effect of physical activity on the improvement of glycemic control has not been demonstrated [7, 8]. On the other hand, exercise can enhance psychological well-being by increasing self-esteem and quality of life [9]. The high risk of hypoglycemia, defined as a glucose level < 3.9 mmol L^-1^ [10], is the greatest barrier to physical activity in patients with type 1 diabetes (T1DM) [11], and fear of it discourages patients [3, 12]. A common suggestion to prevent hypoglycemia is reduction of the insulin dose [13, 14], although the improved glycemic profile is often obtained at the cost of a high glycemia before the effort [15]. The mainstay of exercise management, however, remains an increased ingestion of carbohydrates (CHO) before and during exercise [12, 16–19], especially when exercise is not planned in advance [18, 20, 21], even though an excessive CHO ingestion may result in a detrimental increase of glycemia [21]. An algorithm called *Ecres* (Exercise Carbohydrates Requirement Estimating Software) [18, 22] has been developed with the aim of estimating the amount of carbohydrates patients will need for a particular exercise. Its preliminary application showed that in more than 70% of cases the estimated amounts of CHO allowed patients complete the effort with glycemia within the clinically optimal range [18].

Current guidelines to minimize the risk of exercise-related glycemic imbalances were mainly drawn from exercises lasting up to one hour [14, 17, 18, 22–29]. Nevertheless, in particular when on holiday, patients might be occasionally engaged in long-lasting outdoors leisure-time physical activities (e.g. hiking, hill walking, biking, cross-country skiing), which tend to be light to moderate in intensity. No clear-cut suggestions are available for such activities. Indeed, Grimm et al. [16] suggest a supplement of 30g CHO/h exercise for prolonged exercises (> 1 h) at an intensity < 60% of maximal heart rate, whereas an almost double amount, i.e. up to 60g CHO/h (at the rate of 1g/kg/h), is suggested by others [12].

The present study was performed to investigate the precision and accuracy of the main parameters estimated by the *Ecres* algorithm for prolonged moderate intensity exercise (3-h). To this aim, plasma insulin concentrations, whole-body carbohydrate oxidation rates and required amounts of CHO were measured on a group of patients with type 1 diabetes exercising under free-living conditions. These data were subsequently compared with the corresponding values estimated by the *Ecres* algorithm.

## Materials and Methods

### Participants

Nine T1DM patients (5 men, 4 women; HbA1c 54±13 mmol mol^-1^; 7.1±1.1%;), aged 35–65 years (average 48±11 years) gave their written voluntary consent after having been informed on the nature, purpose, and possible risks. Duration of diabetes and mean daily insulin doses were 28±14 years (range 5–49 years) and 0.52±0.08 IU kg^-1^ day^-1^, respectively. Average body mass and stature were 73±15 kg and 1.70±0.10 m (BMI 25.1±3.0 kg m^-2^), respectively. The study was approved by the Ethics committee of the University of Udine and was conducted according to the Declaration of Helsinki.

Inclusion criteria were no clinical evidence of chronic complications contraindicating physical activity, no medication other than insulin, and self-management skills resulting in an acceptable metabolic balance (HbA_1c_ ≤75 mmol mol^-1^; ≤9.0%). All patients were on a basal-bolus insulin regimen; 5 used insulin lispro before meals and 4 used regular insulin. At bedtime, 8 patients used insulin glargine and 1 used insulin NPH. All the volunteers practiced aerobic physical exercise two to three times a week and none was highly trained.

### Experimental protocol

All the participants were asked to refrain from physical activity, alcohol, and caffeine in the 24 h preceding the test; in addition, they were advised to maintain their usual diet and insulin regimen; a physician checked adherence to usual diet and therapy just before the trials.

All patients arrived at the laboratory at 7:00 AM. Immediately, a care-provider inserted the sensor of a continuous glucose-monitoring device (CGM; Paradigm Revel 523, MedTronic Mini-Med Inc, CA, USA) under the skin of patients' abdominal wall, paying attention to avoid locations that were constrained by clothing or accessories or were subjected to vigorous movement during exercise. According to instructions for use, the CGM device was calibrated 2 h after the sensor was installed and then periodically by uploading a capillary glucose value. Since we are aware that the CGM sensors suffer from low accuracy in their first hours of wear, these data were used only to evaluate the overall trend of glucose levels (in particular during the night following the exercise) with a high time resolution but avoiding unacceptable frequent fingerstick capillary measurements. In addition, an indwelling catheter was inserted into a vein of both forearms, one for blood withdrawals, the other for glucose infusion, if needed; patency of both catheters was maintained by intermittent flushing with saline. At 7:30 AM, patients injected themselves with their usual insulin dose (0.08±0.04 IU kg^-1^) subcutaneously in the abdominal wall and consumed their usual breakfast meal (composed of milk, yogurt, bread or crisp toasts) equating to 0.6±0.2 g kg^-1^ body mass of CHO mainly in the form of starch. The patients’ amount of CHO for breakfast was decreased by 8 g for each 2.8 mmol L^-1^ exceeding 10 mmol L^-1^ of blood glucose concentrations at rest (e.g. for a glucose level of 13.3 mmol L^-1^, the patient received 16 g less carbohydrates compared to his/her usual breakfast).

Until 30 min prior to the start of exercise patients rested in an armchair; at this time patients were equipped with the belt of a heart rate (HR) monitoring system (Polar Electro, Finland) to acquire HR every 15 s throughout the exercise.

Patients performed a 3-h exercise (from 10:00 AM to 1:00 PM) on a treadmill (Saturn, H-P Cosmos, Germany), which automatically adjusted speed to keep constant the target HR. To minimize dizziness due to prolonged treadmill exercise, a 5 min rest was allowed at the end of each hour of exercise. In anticipation of the prolonged exercise, work intensity was set to 30% of the individual HR reserve (i.e. 0.3 x (220—age—HR at rest) + HR at rest), where 220—age (expressed in years) is the estimate of maximal HR. Indeed, during a preliminary test carried out on a group of healthy subjects of similar age, this heart rate was elicited by a walking speed that could be classed as “brisk walk”, i.e. >4.8 km h^-1^ [30]. Consequently, to allow all patients to likely complete the task, higher intensities were not considered adequate. In addition, since the *Ecres* algorithm estimates the required carbohydrates to avoid the exercise-induced glycemic imbalances on the basis of absolute heart rate, in order to mimic a patient’s usual setting, the assessment of patient’s fitness level to set exercise intensity was deemed meaningless.

Oxygen consumption, carbon dioxide production, and ventilation were measured by means of a metabolic unit (Quark b2, Cosmed, Italy) during the 10 central minutes of each half-of-an-hour period of the exercise.

Venous blood (5.5 mL) was drawn at rest (at 7:30 AM), 1 hour before the start (at 9:00 AM), at the start and thereafter every 30 min until the end of the exercise. A drop of blood was immediately used to test blood glucose concentration using reactive strips (ContourLink, Bayer Healthcare Diabetes Division); the same strips were used to test glycemia by finger-stick capillary blood when the CGM device showed a fall in glucose concentration.

To avoid hypoglycemia, on the basis of the expertise of the same physician attending the whole experimentation (skilled in prevention of exercise-induced hypoglycemia), before the start of exercise patients were administered known amounts of fruit fudge (Perugina, Italy; 93% sucrose, one candy weighing 7.5 g). The number of administered candies depended on the measured glucose level and ranged from zero (if glycemia was >10.0 mmol L^-1^) to 2 candies (when glycemia was <6.7 mmol L^-1^). Additional candies were administered for each of the following 30 min exercise periods, on the basis of the change in glycemia observed during the preceding period. Furthermore, in the case glycemia fell below 5.0 mmol L^-1^ during the last 10 min of the half-an-hour of exercise period, known volumes of 33% w/v glucose solution were infused to restore a safe glycemia. We are aware that patients cannot apply such a strategy under usual life conditions; nevertheless, it allowed us summing up all the amounts of administered CHO within the 30 min periods avoiding the uncertainty related to the sucrose absorption rate. All patients were allowed to drink water ad libitum.

At the end of the exercises, to avoid severe late-onset hypoglycemia, defined as hypoglycemic events occurring within 15 h after the exercise [19, 31], patients were kept under supervision of the physician until the following morning, when the CGM sensor was removed (at 7:00 AM). Indeed, the pre-exercise muscle glycogen level is almost completely restored after 12 hours recovery [32], although physical activity can affect insulin sensitivity up to 48 hours [33]. During the observation period, patients maintained their usual insulin doses and meals and all the glycemic levels measured on capillary blood or provided by the CGM device showed that no late-onset hypoglycemia occurred.

### Calculations

At the end of the exercise, HR data were averaged offline over the 30 min periods. Oxygen consumption ($\dot{\text{V}}{\text{O}}_{2}$) and carbon dioxide production ($\dot{\text{V}}\text{C}{\text{O}}_{2}$) data were averaged over the corresponding 10 min recording periods. These data allowed calculating the respiratory quotient, assumed to be equal to the respiratory exchange ratio (RER). Hence, by assuming that proteins do not contribute to any significant extent to the energy production during exercise [34], the whole-body fat and carbohydrates (CHOox) oxidation rates and the energy expenditures were computed [35].

Actual CHO requirements for each 30 min exercise period were calculated by summing up all the CHO administered in the form of fruit fudge and the amount of infused glucose.

Finally, using the *Ecres* algorithm (see below), insulin concentrations and whole-body carbohydrate oxidation rates were estimated a posteriori as well as the amounts of CHO required for each half-an-hour exercise. To this aim, patient’s specific diet and therapy were uploaded in the algorithm as well as actual heart rate during the exercise (averaged over 30 min periods) and the glucose levels determined by means of the reactive strips before the start of the exercise and at 30 min intervals.

### The *Ecres* algorithm

The *Ecres* algorithm consists of two procedures, briefly described here. In the first one (a formerly set-up procedure), patient’s usual therapy (i.e. types, doses and time scheduling of all the insulin administrations together with usual dietary carbohydrates) and individual’s fitness level (“sedentary” or “active”) are loaded in the system. The algorithm estimates the plasma insulin concentration profile throughout the day on the basis of the uploaded usual therapy and on ‘‘standard” pharmacokinetic profiles of the commercial insulin preparations. Subsequently, taking into account patient’s insulin sensitivity (computed as patient’s usual dietary carbohydrates to insulin ratio for each day period, i.e. morning, afternoon or evening), and applying the linear relationship between percentage of carbohydrates and insulin concentration previously described [36], the algorithm estimates the daily profile of the fractions of overall amount of CHO burned during the exercise (totCHOox) that are required to avoid glycemic imbalances. This first procedure needs to be run at the start of the use of the algorithm and, subsequently, only when relevant changes of the patient’s usual therapy and/or diet are introduced.

The second procedure is run at each exercise occasion and requires only the entering of expected average HR and exercise duration, together with actual glycemia. Occasional changes of the usual therapy (either of the time scheduling of the last injection and/or of the insulin dose) might also be introduced for appropriate corrections. The algorithm computes the totCHOox as the product of exercise duration and whole-body carbohydrates oxidation rate (CHOox), in turn estimated on the basis of the expected HR (index of exercise intensity) and the patient’s fitness level [37]. Since healthy subjects show a decrease in CHOox with increasing exercise duration [38], for the present investigation, the overall exercise duration (3 hr) was subdivided in 6 consequent periods lasting 30 min each and the estimated CHOox were arbitrarily reduced by 8, 16, 23, 30 and 34% for the second to the sixth period, respectively [38]. The CHO requirement is calculated as the appropriate fraction of totCHOox, according to the time of day the exercise is performed. Finally, the excess or lack of glucose contained in the extra cellular compartment is computed as the product between the extra cellular fluid volume (ECF) and the difference between actual glycemia, as measured by means of reactive strips, and the target glucose level recommended for T1DM patients at the same time distance after a meal [39]; this quantity is subtracted from the estimated CHO requirement.

### Analyses

A drop of the blood drawn was used to immediately test glycemia (ContourLink, Bayer Healthcare Diabetes Division). The remaining blood was divided into a 2 mL Vacutainer tube (#368920) containing a glycolysis inhibitor (4 mg of kalium oxalate + 5 mg of sodium fluoride) and a 3.5 mL Vacutainer tube (#368965) with gel and clot activator. Immediately thereafter, both the tubes were gently inverted and stored at +4°C until the hospital laboratory centrifuged the samples to separate serum and perform the measurements.

Plasma glucose concentration was determined by applying a hexokinase-based methodology (Olympus Diagnostic Systems AU2700) (coefficient of analytical variation was <2% in the range of 3.27–11.67 mmol L^-1^). Insulin concentrations were assayed by the DxI800 Automated Immunoassay system (Beckman Coulter, CA, USA) (coefficient of analytical variation was <6% in the range of 73.9–327.5 pmol L^-1^). This insulin assay measures all the relevant insulin analogs showing a cross-reactivity of about 80% [40].

### Statistical Analysis

The data were analyzed using standard statistical methods (Systat vs.11). Two-way analysis of variance with repeated measures design (MANOVA, with time as within-subjects factor) was applied, followed by specific contrasts when appropriate. If the sphericity assumption appeared to be violated, the degrees of freedom were adjusted using the Greenhouse–Geisser ε. The Pearson correlation coefficient was used to look for associations between the study parameters. A p value of <0.05 (two-tailed) was assumed as statistically significant. Data are expressed as means ± SD.

## Results

Average actual heart rate during the 3-h exercises amounted to 101±6 bpm (i.e. 29.8±3.7% of individual’s HR reserve); it reached the target value in a few minutes, and thereafter remained stable over time (time effect, F = 1.36, *P* = NS). Progression speed remained stable throughout the exercise (time effect, F = 1.55, *P* = NS), amounting on average to 1.42±0.14 m s^-1^ (i.e. 5.1±0.5 km h^-1^). Average $\dot{\text{V}}{\text{O}}_{2}$, expressed per unit body mass, amounted to 13.3±3.2 mL min^−1^ kg^−1^ and did not change significantly throughout the exercise (time effect, F = 1.63, *P* = NS), resulting in an energy expenditure of 20.0±7.4 kJ min^−1^.

**Fig 1:**
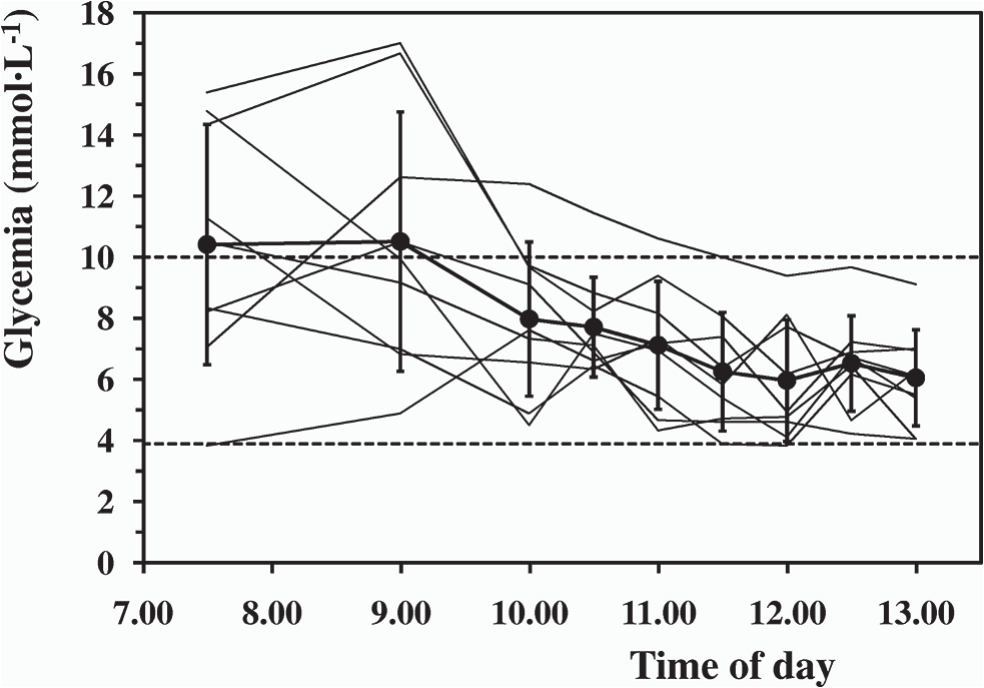
Blood glucose levels throughout the trials. Average glycemia (dots and thick line) throughout the trials is plotted against day-time; thin lines are the individual blood glucose levels at rest, before and throughout the 3-h exercise are also illustrated. Horizontal dashed lines: hypoglycemic and hyperglycemic thresholds.

Basal glucose concentration (at 7:30 AM) amounted on average to 10.4±3.9 mmol L^-1^ and remained substantially stable for about 90 min. During the exercise, glucose concentrations decreased significantly (from 8.0±2.5 mmol L^-1^ to 6.1±1.6 mmol L^-1^; time effect, F = 6.76, P<0.001). Individual plasma glucose levels throughout the trials are illustrated in Fig 1.

**Fig 2:**
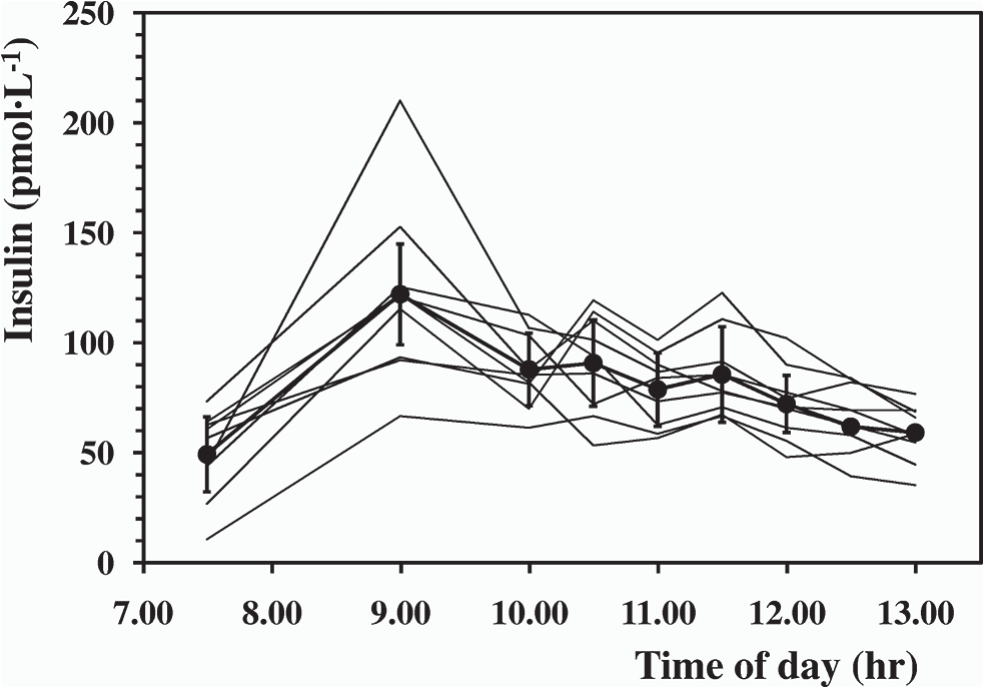
Plasma insulin concentrations throughout the trials. Average laboratory measured plasma insulin concentrations (full dots and thick line) at rest, before and throughout the 3-h exercise is plotted against day-time; thin lines are the individual concentrations.

**Fig 3:**
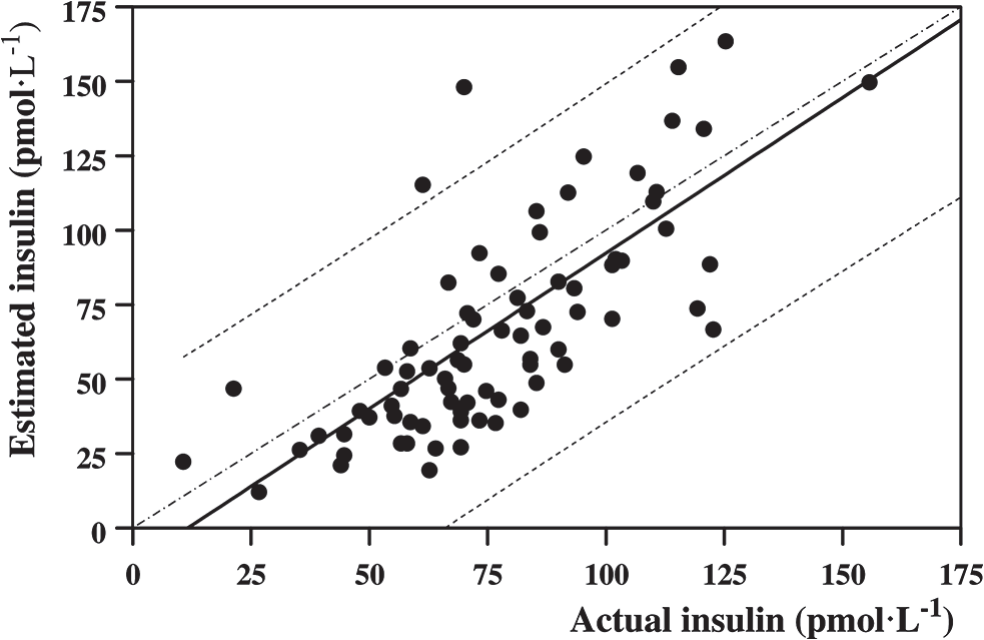
Relationship between insulin concentrations estimated by the *Ecres* algorithm and the corresponding laboratory-measured values. The relationship between estimated and measured insulin concentrations was statistically significant (R = 0.771, p < 0.001, n = 81). Dashed lines are the 95% Confidence Limits. Dashed-dotted line is the identity line.

Laboratory measured basal insulin concentrations (before breakfast) amounted on average to 50.2±20.8 pmol L^-1^; they peaked at 9:00 AM (135.3±46.0 pmol L^-1^) and thereafter fell significantly to 59.1±13.0 pmol L^-1^ (time effect, F = 8.20, *P*<0.001) (Fig 2). The corresponding insulin concentrations estimated by the *Ecres* algorithm at the same daytimes of the blood withdrawals amounted to 30.8±13.3 pmol L^-1^, 150.1±50.8 pmol L^-1^ and 41.6±10.4 pmol L^-1^, respectively; overall, estimated insulin concentrations were not statistically different from the laboratory-measured values (type effect, F = 2.19; *P* = NS). The estimated insulin concentrations (pmol L^-1^) were significantly related to the measured values (pmol L^-1^), as described by (Fig 3):

Estimated insulin=1.04x Measured insulin−12.2

(R = 0.771, *P* < 0.001, n = 81).

**Fig 4:**
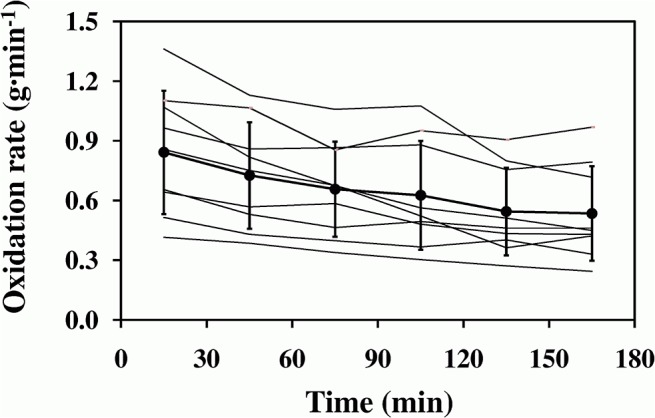
Whole-body carbohydrates oxidation rates throughout the exercise. Average whole-body carbohydrates oxidation rates (full dots and thick line) are illustrated as a function of exercise duration. Thin lines are the individual oxidation rates.

**Fig 5:**
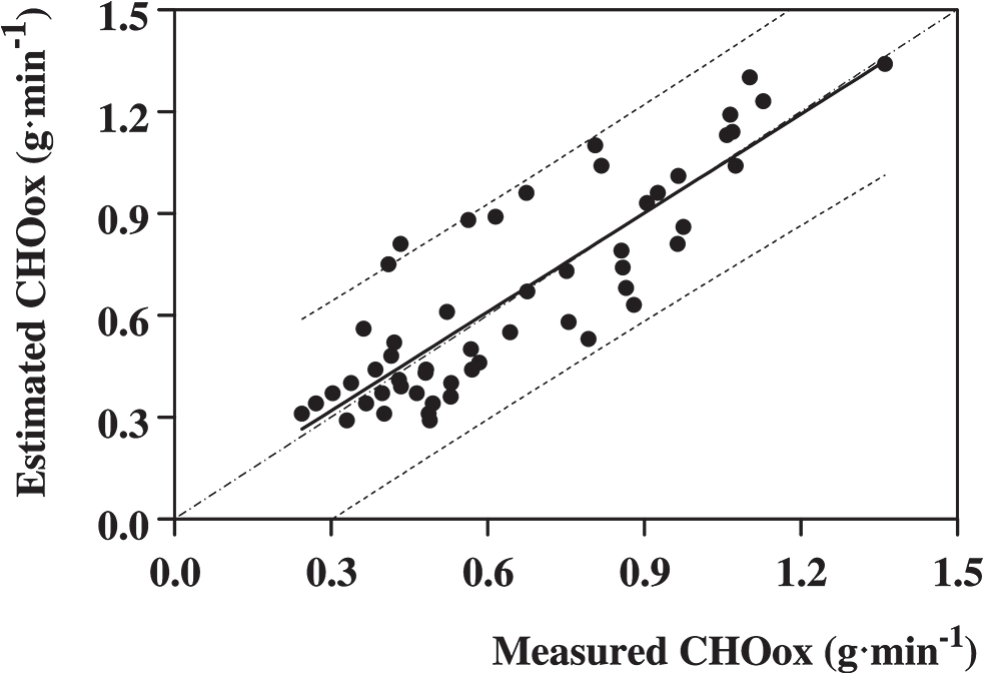
Comparison between the estimated carbohydrates oxidation rate and the corresponding actually measured values. Relationship between the estimated carbohydrates oxidation rates over 30 min period of the exercise and corrected for the overall exercise duration, and the actually measured values. The relationship was statistically significant and close to the identity line (R = 0.877, p < 0.001, n = 54). Dashed lines are the 95% Confidence Limits. Dashed-dotted line is the identity line.

Across the exercise, the measured CHOox (g min^-1^) decreased significantly with time from 0.84±0.31 g min^-1^ to 0.53±0.24 g min^-1^ (time effect, F = 10.64, P<0.001) (Fig 4), whereas fat oxidation increased significantly from 0.20±0.09 g min^-1^ to 0.28±0.14 g min^-1^ (time effect, F = 3.48, P<0.01). The corresponding estimated CHOox amounted to 0.81±0.37 g min^-1^ and 0.53±0.24 g min^-1^, respectively; overall, estimated CHOox were not significantly different from the actually measured ones (type effect, F = 0.86, P = NS). The two quantities were significantly related to each other (Fig 5), as described by:

Estimated CHOox=0.995x Measured CHOox+0.01

(R = 0.877, *P* < 0.001, n = 54).

**Fig 6:**
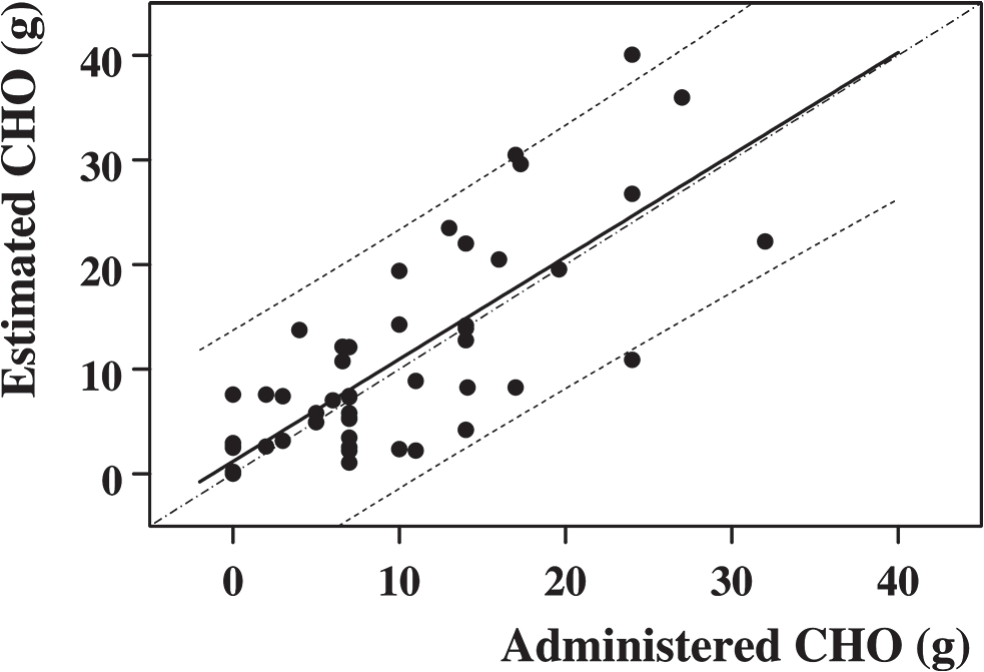
Comparison between the estimated amounts of required carbohydrates and the corresponding actually administered quantities. Relationship between the amounts of required carbohydrates estimated by the algorithm for the 30 min exercise periods and the actually administered quantities. The relationship was statistically significant and close to the identity line (R = 0.788, p < 0.001, n = 54). Dashed lines are the 95% Confidence Limits. Dashed-dotted line is the identity line.

For each half-an-hour of exercise the carbohydrates requirement amounted to 8.9±7.9 g (range 0–32 g), independent of the elapsed time (time effect, F = 0.43, *P* = NS). Required carbohydrates estimated by the *Ecres* algorithm amounted to 9.9±9.8 g (range 0–40 g) and were not significantly different from the actual administered ones (type effect, F = 0.49, *P* = NS). The two quantities were significantly related to each other (Fig 6), as described by:

Estimated CHO=0.977x Administered CHO+1.19

(R = 0.788, *P* < 0.001, n = 54).

**Fig 7:**
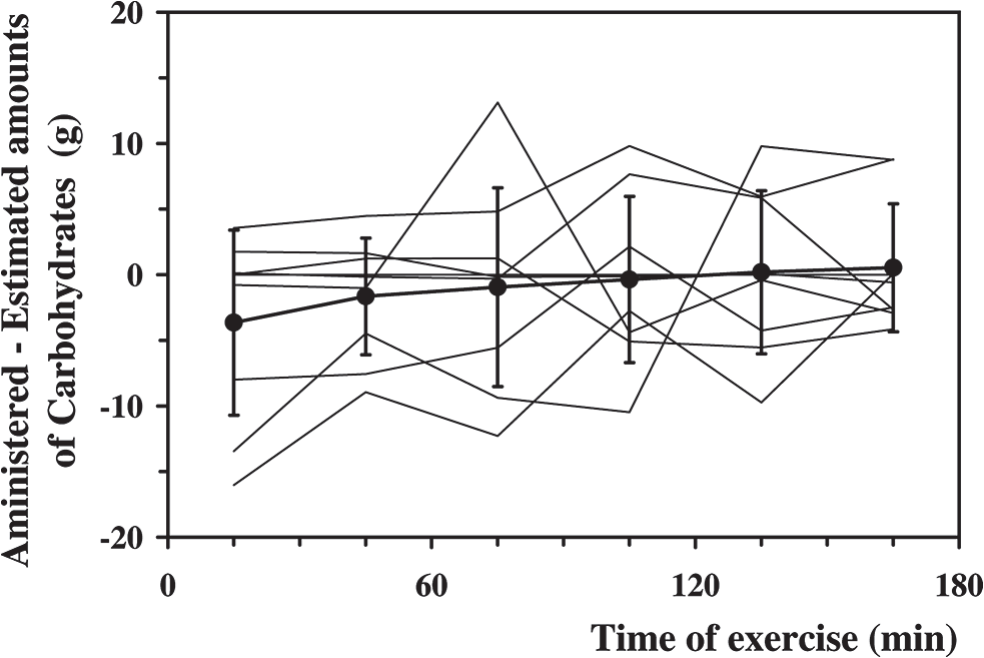
Individual differences between the estimated amounts of carbohydrates and the actually administered quantities. Average differences (full dots and thick line) between the actually administered amounts of carbohydrates for each of the 30 min exercise periods and the corresponding quantities estimated by the *Ecres* algorithm. Thin lines are the individual differences.

Average difference between them amounted to—1.0±6.1 g, independent of the elapsed time (time effect, F = 0.82, *P* = NS). The individual differences between actually administered amounts of carbohydrates and estimated quantities are illustrated in Fig 7.

## Discussion

Main results of the present work show that the *Ecres* algorithm estimates adequately the amounts of carbohydrates required by T1DM patients to minimize the risk of exercise-induced glycemic imbalances (mainly hypoglycemia) also during prolonged moderate intensity physical activity [30, 41]. Indeed, while glucose concentration was kept in the clinically optimal range [42] throughout the whole exercise, the estimated amounts of carbohydrates were close to the actually administered ones (fruit fudge + infused glucose), showing an average difference of—1.0±6.1 g.

The suggestions of Grimm et al. [16] quote an amount of 30 g CHO/h for an intensity <60% of maximal heart rate and prolonged activity (> 60 min); this quantity would have been excessive as compared to the actual requirements of the present investigation, resulting in a difference of 6.1±7.9 g. An even worse result would be obtained with the suggestion of 1 g CHO/kg/h [12]. It can thus be concluded that, although the *Ecres* algorithm did not estimate the CHO requirements for the prolonged exercise with full accuracy, it provided better suggestions as compared to those available from the literature.

Results are in agreement and integrate previous work [18, 22] showing that the *Ecres* algorithm estimated adequately the supplemental CHO required by a T1DM patient to avoid immediate exercise induced glycemic imbalances in about 70% of 1-h duration moderate intensity exercises, independent of the time of day the activity was performed.

Indeed, a reduction of the pre-meal insulin dose is often suggested [12, 14] to minimize the risk of exercise-induced hypoglycemia. This strategy, however, can be applied only when exercise is foreseen and is helpful when the exercise is planned in the post-prandial hours (e.g. within 2 hours from the meal). Moreover, a drawback of this strategy is that the improved glycemic profile during exercise is usually obtained at the cost of a high glycemia before the effort [15]. Although additional CHO ingestion results in an increased caloric intake, this strategy before and during exercise remains the mainstay of exercise management in T1DM patients [12], in particular when exercise is not planned in advance [18], or in patients on multiple daily insulin injections exercising at longer time distances from the meals.

### The whole-body carbohydrate oxidation rate

A time-dependent decrease in the CHOox during the 3-hr exercise was observed in our T1DM patients, even though they were administered fruit fudge to avoid an excessive fall of glycemia. Indeed, the carbohydrate oxidation rate and its time-dependent decrease were very similar to those observed in healthy people [43, 44]. Present results confirm that an elevated extracellular glucose availability accompanied by a high insulin level, a frequent condition in T1DM patients, does not necessarily translate into a different behavior of CHOox as compared to healthy people [44, 45].

The estimated CHOox during the 3-h exercise were linearly related to the actually measured ones, the relationship lying close to the identity line, suggesting that the *Ecres* algorithm estimated adequately the whole-body carbohydrates oxidation rate. The main cause of the difference between estimated and actually measured values might be the assignment of patients to one out of only two possible fitness levels (i.e. “sedentary” or “active”), neglecting possible intermediate fitness levels which likely might ameliorate the estimates of the algorithm. In many instances, however, the difference between estimated and actual CHOox might be negligible [18, 22], since the prevailing insulin concentration during exercise sets the proportion between the amount of oxidized carbohydrates and the amount of carbohydrates required to avoid exercise-induced glycemic imbalances [36].

### The blood insulin concentration

The behavior of the laboratory determined insulin concentrations was compatible with the average pharmacokinetic profile of the insulin preparations used by patients before breakfast (at 7:30 AM). The linear relationship observed between the measured and the estimated insulin concentrations, showing a slope close to 1 and a quite small intercept, suggests that the *Ecres* algorithm provides rather good estimates of the actual insulin concentrations. This result is very intriguing, in particular taking into account the difficulties inherent to the determination of the recombinant insulin analogues using automated clinical laboratory analyzers [40, 46].

### Strengths and limitations of the study

The demanding protocol (i.e. 3-h exercise, followed by the need of surveillance by an expert physician until the following morning because of the high risk of late-onset hypoglycemia) made it hard to recruit a wider number of participants. In addition, we are aware that the variety of patients included (as concerns e.g. age or insulin requirements) inevitably has introduced some noise in the acquired data. Nevertheless, we believe that the analyses carried out by subdividing the overall exercise duration in periods lasting 30 min yielded a relevant number of data (n>54), which provided interesting and new information.

Patients were studied without changing either their usual insulin dose or clamping their glucose or insulin levels, thus simulating an unexpected physical activity under free-living conditions. Since heart rate monitoring is the most common, efficient and economical means that people use to check exercise intensity, HR was held constant throughout the exercises. Indeed, a constant HR does not fully replicate possible speed and/or gradient changes of a real walk that might alter the HR response. The carbohydrates requirements, however, were estimated for 30 min exercise sub-periods and could be easily changed by using different HRs for subsequent periods. Finally, the selected target HR (i.e. 30% of HR reserve) might seem low. Nevertheless, actual heart rate corresponded to about 58% of patients’ maximal heart rate and progression speed was similar to the average normal gait speed of healthy subjects of similar age [47]. Preliminary test made us confident that under these conditions all patients could complete the task, whereas higher work rates likely would decrease the compliance of participants.

Only sucrose, in the form of sugar drops, was administered to patients to counteract the possible fall of glycemia. Different types of ingested CHO (e.g. starches) or the contemporary consumption of other nutrients (e.g. fats or proteins) might likely result in a delayed glycemic response; under these conditions, the risk of hypoglycemia can be expected to remain high until the ingested glucose is fully assimilated, whereas glycemia after the end of exercise might rebound to excessively high values.

A further limitation is that all the patients performed the exercise during the morning. The performance of the *Ecres* algorithm for prolonged exercises performed in the afternoon should also be tested; indeed, patient insulin sensitivity likely changes and the algorithm might perform a worse estimate of it [18, 22]. Transitory changes of the patient’s usual insulin sensitivity during the trials, which likely resulted in a correspondent change in the carbohydrates requirement for the exercise, might explain, at least in part, the discrepancies observed between the actually administered amounts of carbohydrates, established by an expert physician attending the whole experimentation, and the estimated quantities. Undoubtedly, the introduction in the algorithm of an appropriate correction for temporary changes in patient’s insulin sensitivity might be a future intriguing challenge.

Several different implementations of the algorithm can be foreseen, ranging from simple software to specific devices, which can be easily integrated in training equipments or glucose monitoring systems. In addition, a real-time version of the algorithm can be foreseen, where the residual supplemental CHO still available during exercise will be estimated on the basis of actual HR, averaged over short time intervals, unfolding the prospect of a very intriguing device able to warn patients of the concrete risk of hypoglycemia. Although the algorithm was developed so far only for patients on multiple daily insulin injections, specific features for insulin pump users can be easily integrated.

## Conclusions

Results confirm that, also during prolonged exercise, the *Ecres* algorithm provides a satisfactory estimate of the main parameters affecting exercise metabolism in T1DM patients, resulting in appropriate amounts of carbohydrates required to maintain glycemia within the clinically desired limits.

Use of the *Ecres* algorithm will liberate patients from the need of understanding the metabolic responses to exercise, thus minimizing risk of harm and reducing their fear of hypoglycemia while maximizing their pleasure for exercise.
